# Allogeneic platelet-derived growth factors local injection in treatment of tennis elbow: a prospective randomized controlled study

**DOI:** 10.1007/s00264-022-05300-9

**Published:** 2022-01-12

**Authors:** Mahmoud Ibrahim Kandil, Abdel-Salam Abdel-Aleem Ahmed, Rasha Shaker Eldesouky, Sherif Eltregy

**Affiliations:** 1 Department of Orthopaedic Surgery, Benha Faculty of Medicine, Farid Nada Street, Kalyubia, Banha, Post Office, 13518 Egypt; 2 Community Medicine, Faculty of Medicine, Benha University, Banha, Egypt

**Keywords:** Tennis elbow, Allogeneic growth factors, Lyophilized human platelets growth factors (L-GFs), qDASH, PRTEE

## Abstract

**Purpose:**

The purpose of this study aimed to evaluate the efficacy of local injection of allogeneic platelet-derived growth factors in treatment of patients with tennis elbow.

**Patients and methods:**

This study included 120 tennis elbow patients randomly divided into two groups. The patients were locally injected with allogeneic growth factors (treatment group) or with normal saline (control group). The outcomes were assessed using Patient-Related Tennis Elbow Evaluation (PRTEE) and quick Disabilities of the Arm, Shoulder and Hand (qDASH) scales. The clinical outcomes were accordingly classified as excellent, good and poor. The patient’s satisfaction and adverse effects were also recorded.

**Results:**

There was no statistically significant difference between the two groups regarding the age, gender, dominant arm or the pre-injection scores. At three month follow-up, the reductions in the mean PRTEE and qDASH scores were 88.7% and 70.6% in the treatment group versus 21.8% and 14.9% in the control group, respectively. At the last follow-up, the outcomes in the treatment group were excellent in 85% of patients and good in 15%, versus 8% and 32% in the control group. Overall, 95% were satisfied in the treatment group compared to 25% in control group. Forty patients in the treatment group experienced mild transient post-injection pain.

**Conclusion:**

This study strongly suggests that local injection of allogeneic platelet-derived growth factors could be a promising safe treatment option for tennis elbow with significant pain relief, functional improvement and patient’s satisfaction. Yet, additional larger studies are needed to assess the durability of these outcomes.

## Introduction

Tennis elbow is one of the most common overuse syndromes in the upper extremity that predominantly involves the origin of short radial extensor muscles of the wrist [1]. It is believed to be due to angio-fibroblastic and mucoid degenerative processes affecting this tendinous origin secondary to failure of natural tendon repair mechanism, after repeated strenuous activities, rather than a mere inflammatory process [2]. It is a self-limited disease with tendency to natural resolution [3, 4]. Despite variable treatment options, there is no consensus on a single treatment method with consistent efficacy [5–8].

Allogeneic platelet-derived growth factors injection was reported for treatment of plantar fasciitis and knee osteoarthritis [9, 10]. They include platelet-derived growth factor (PDGF), transforming growth factor beta (TGFS-β), epidermal growth factor (EGF), vascular endothelial growth factor (VEGF) and insulin-like growth factor [1, 2, 11, 12]. These factors increase wound, bone and tendon healing through promoting cell migration, proliferation, differentiation, extracellular matrix synthesis and angiogenesis [12–14]. They were reported to be beneficial in treating tendinopathies, with a potential to reverse the degenerative changes and promote regeneration of tendinous tissues [15, 16].

Unlike PRP preparation, the allogeneic platelet-derived growth factors are derived from pathogen-free platelets from other individuals within the same species rather than from autologous platelets [17]. They undergo a process of lyophilization (freeze drying) to stabilize the biologic materials making them suitable for prolonged storage without significant change in biologic structure or efficacy [18].

The lyophilized growth factors (L-GF) vial is a preparation containing lyophilized human allogeneic platelet-derived growth factors. It has growth factors concentration equivalent to an autologous PRP preparation obtained from 20 mL of whole blood with a platelet count of 106/μL, but with a much longer shelf life (12–18 months versus only 8 hours). It is suitable for local intralesional injection being a water-soluble product with no gel formation [9, 10].

The purpose of this prospective randomized controlled study was to evaluate the efficacy of local injection of allogeneic platelet-derived growth factors (L-GF vial) compared with placebo injection in patients with tennis elbow.

## Patients and methods

This prospective randomized controlled clinical trial was done between May 2017 and January 2020 at the orthopaedic department of our University Hospital, after approval of the Research Ethics Committee at Faculty of Medicine, University (REC-FOMBU). The preparation of this randomized controlled study followed the guidelines of the Consolidated Standards of Reporting Trials Group (CONSORT Group) [19].

Skeletally mature patients with tennis elbow were included in this study. Exclusion criteria included patients with systemic disorders (e.g. anaemia, coagulation disorders, DM, hepatitis or rheumatoid arthritis), local elbow conditions (previous local corticosteroid or PRP injections, arthritis, previous trauma or surgery, nerve entrapment, infection or malignancy), cervical spine pathology, psychiatric disorder or pregnancy. The diagnosis was made by pain and tenderness over the lateral aspect of the elbow, and two of the following tests being positive: wrist extension (Cozen’s test), Mill’s manoeuvre, jar lifting test, wringing test, broom test or stir-frying test. All patients had dissatisfaction with symptoms six weeks after a first specialty visit (ranged from 6 to 10 weeks with an average of 7.3). The conservative treatment included oral and topical NSAIDs and tennis elbow brace. All patients had complete physical examination, laboratory investigations (as complete blood count, glucose level, ESR, C-reactive protein, bleeding profile, serum uric acid and rheumatoid factor) and imaging studies (orthogonal elbow and cervical spine radiographs).

**Fig. 1:**
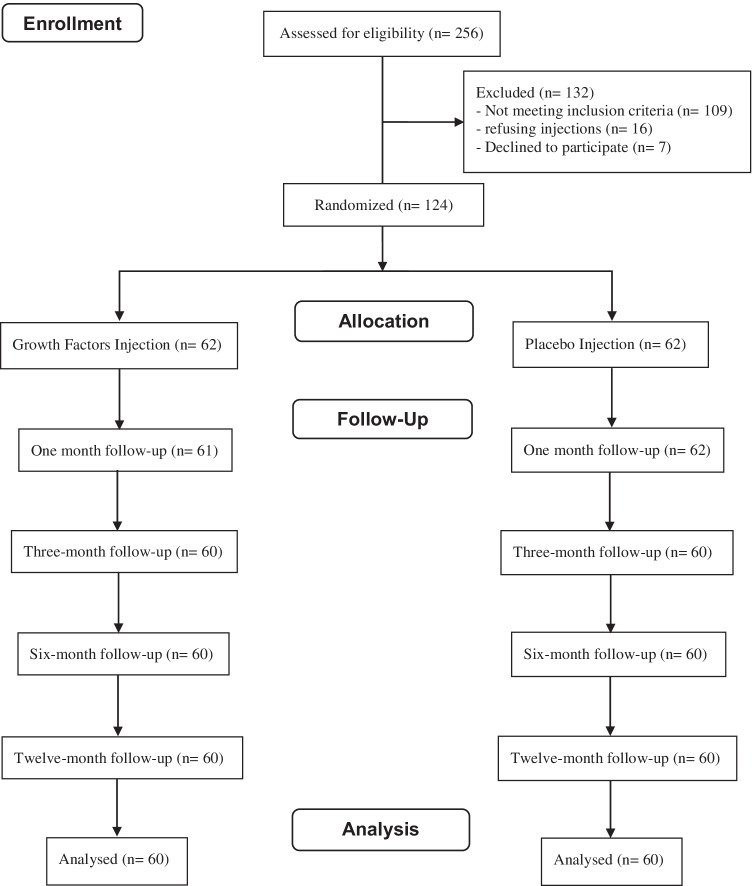
A chart detailing the patient flow in the study

An informed consent was obtained from all patients after giving detailed information about the study. During the study period, potentially eligible patients who met the inclusion criteria were 147 of 256 tennis elbow cases. Twenty-three cases of the 147 were excluded for refusing injections (16 patients) or not accepting inclusion in a research study (seven patients). Thus, 124 patients were ultimately enrolled and randomly assigned to one of two groups. The treatment group included 62 patients injected with L-GF. The control group included 62 patients injected with normal saline. Four patients (two in each group) did not complete follow-up and consequently were excluded from data analysis with eventual assessment of 60 patients in each group (Fig. 1). The randomization process was done through sealed opaque envelopes where the allocation group was stated. This study was single-blinded where only the patients were blinded to their treatment assignment.

The baseline patients’ demographics are outlined in Table 1. The study included 77 males (64%) and 43 females (36%). The mean age was 36.55 years (range: 23–61). The dominant arm was affected in 91 patients (76%).

**Table 1:** Baseline patients’ criteria

Variables	Treatment group (*n* = 60)	Control group (*n* = 60)	*P* value
Age (y)			
Mean (SD) Range	33.72 (8.49) 23–56	39.38 (5.37) 28–61	.89
Gender,* n* (%)			
Male Female	34 (57) 26 (43)	43 (72) 17 (28)	.128
Dominant arm, *n* (%)	49 (81)	42 (70)	.56
PRTEE (pre-injection)			
Mean (SD) Range	90 (7.49) 85–100	93 (7.23) 90–100	.85
qDASH (pre-			
injection) Mean (SD) Range	78.64 (6.8) 65.9–90.9	77.73 (8.94) 52.3–86.4	.79
Follow-up (*m*)			
Mean (SD) Range	13.56 (1.2) 12–15	14.76 (2.08) 13–17	.74

*n*, number; *SD*, standard deviation; *y*, year; *PRTEE*, Patient-Related Tennis Elbow Evaluation; *qDASH*, quick Disabilities of the Arm, Shoulder and Hand; *m*, month

The L-GF vials contain lyophilized human platelets growth factors derived from individual whole blood donations. The preparations were tested for hepatitis B surface antigen, hepatitis C virus antibodies, HIV I and II antibodies, HIV p-24 antigen and *Treponema pallidum* antibodies. Viral inactivation was performed by ultraviolet radiation and riboflavin using a pathogen-reduction technology system (Mirasol system; Terumo BCT, Inc.). The platelets were in vitro activated with subsequent release of the growth factors followed by a process of ultra-concentration, and finally lyophilization. The L-GF vials are supplied as powder in a sterile sealed container. The vial content is mixed with 3 mL sterile water and allowed to stand at ambient temperature for five minutes before injection (being stored at 2–8 °C before usage).

### Technique of injection

The local injection technique was the same in all patients. The patients were blinded to the injectable solution. The syringe was filled away from the patient and wrapped with aluminium foil to hide the colour of the solution from the patient. Determining the maximum tender area was done by palpating the lateral aspect of elbow region. Under complete aseptic condition, injection was done with single skin entry and multiple pricks in the tendon of extensor carpi radialis brevis. Patients were injected with 3 mL of allogeneic growth factors in the treatment group and 3 mL of normal saline 0.9% in control group using an 18-gauge needle.

### Post-injection protocol

Patients were advised to rest in supine position with elbow flexed 90° for 15 to 20 minutes after injection. Thereafter, the affected elbow was supported by elastic crepe bandage and collar and cuff arm sling for two to three days. NSAIDS were avoided for ten days before and after injection. Paracetamol 500-mg tablets could be given during that period with local ice application. After ten days, stretching exercises of the wrist extensors were performed for the next ten days, followed by formal strengthening program for another ten days. In one month post-injection, recreational activities as tolerated were allowed. Heavy activities (especially that involve wrist and fingers extension) and lifting heavy objects were not allowed for three months.

### Assessment of the outcomes

All the patients were evaluated by Patient-Related Tennis Elbow Evaluation (PRTEE) and quick Disabilities of the Arm, Shoulder and Hand (qDASH) scales. The assessment was done pre-injection and at one, three, six and 12-month post-injection. The PRTEE scale is a 15-item questionnaire to measure the level of pain and disability related to tennis elbow (five items for pain, six items for specific activities and four items for usual activities). The total score is between 0 and 100% where lower score indicates lower level of pain and disability [20]. The qDASH scale is a shortened version of the DASH Outcome Measure that uses 11 items (instead of 30) to measure the physical function and symptoms in any patient with musculoskeletal disorders of the upper limb. The total score is between 0 and 100%, and lower score correlates with lower level of disability and better function [21]. As in other studies, successful results were defined as reduction of 25% or more of PRTEE and qDASH scores in any group at the last follow-up [22, 23].

The results were also evaluated through clinical assessment and were classified as excellent, good and poor (Table 2). Finally, the patients were questioned about their satisfaction and classified into completely satisfied, satisfied with some or important reservations and dissatisfied. Any adverse effect or complication related to the procedure was also recorded.

**Table 2:** The clinical outcome assessment

Outcome	Pain	Tenderness	Cozen’s test
Excellent	No	No	** − **Ve
Good	No	Deep	** − **Ve
Poor	May be	Superficial	** + **Ve

### Statistical analysis

Data were analysed using SPSS version 22.0 software (IBM Corp., Armonk, NY, USA). A power analysis was performed using the MedCalc program ((MedCalc Software, Mariakerke, Belgium) to determine the least sample size required to test a significant difference of DASH scores between the groups (the effect size that would be significant, 13 points) based on the standard deviations obtained from a previous study on patients with lateral elbow pain [8]*.* It was found that 48 patients in each group would provide 90% statistical power at a 5% level of significance. To account for a possible loss to follow-up of 20 to 25%, the number was increased to 62 participants in each group.

Categorical data were presented as number and percentages and analysed by Chi square test or Fisher’s exact test. Continuous variables were tested for normality by Kolmogorov–Smirnov test, assuming normality at *P* = 0.05. Quantitative data were presented as mean ± SD and analysed by Student “*t*” test for 2 independent groups. Paired samples over the period of the study were analysed by repeated measures ANOVA, with pairwise comparisons by the adjusted paired “*t*” test. Two-sided *P* ≤ 0.05 was stated significant.

## Results

The study included 120 unilateral tennis elbow patients with a mean follow-up period of 13.56 months for treatment group and 14.76 months for the control group. There was no statistically significant difference between the two groups regarding age, gender or percent of dominant arm affection. None of the patients received any crossover or additional treatment such as additional injections, therapy and procedures in the follow-up period.

At baseline, there was no statistically significant difference between the two groups regarding the mean PRTEE (90 in treatment group versus 93 in control group, *P* = 0.85) and qDASH scales (78.64 in treatment group versus 77.73 in control group, *P* = 0.79). The primary efficacy endpoint was the change in the pre-injection PRTEE and qDASH scales recorded at three month follow-up (Table 3). At three month follow-up, the reduction in the mean PRTEE score was 88.7% in the treatment group (from 90.5 to 10.2) and 21.8% in the control group (from 93.7 to 73.2) (*P* < 0.001), whereas the reduction in mean qDASH score was 70.6% in the treatment group (from 78.6 to 23.1) and 14.9% in the control group (from 77.7 to 66.1) (*P* < 0.001). At 12-month follow-up, the mean PRTEE score was 10.8 in the treatment group and 69.6 in the control group, whereas the mean qDASH score was 24.5 in the treatment group versus 54.1 in the control group.

**Table 3:** Changes in the mean PRTEE and qDASH scales during the follow-up period

Variable	Mean PRTEE score		*P* value	Mean qDASH		*P* value
	Treatment group (mean ± SD)	Control group (mean ± SD)		Treatment group (mean ± SD)	Control group (mean ± SD)	
Pre-injection	90 ± 7.49	93 ± 7.23	0.85	78.6 ± 6.8	77.7 ± 8.94	0.79
At 1-month	33.7 ± 6.5	89.1 ± 12.3	< 0.001	42.8 ± 8.9	78.2 ± 11.3	< 0.001
At 3-month	10.2 ± 2.4	73.2 ± 9.9	< 0.001	23.1 ± 8.6	66.1 ± 12.1	< 0.001
At 6-month	9.5 ± 1.6	75.4 ± 6.9	< 0.001	24 ± 5.6	60.2 ± 7.9	< 0.001
At 12-month	10.8 ± 2.0	69.6 ± 7.2	< 0.001	24.5 ± 6.0	54.1 ± 9.4	< 0.001
P _Repeated measures ANOVA_	˂0.001			˂0.001		
Paired difference^*^ (mean ± SD)	80.3 ± 8.7	20.5 ± 3.9	< 0.001	55.5 ± 6.3	11.6 ± 1.8	< 0.001
Percentage of score reduction^**^ (mean ± SD)	88.7 ± 9.5	21.8 ± 4.4	< 0.001	70.6 ± 8.1	14.9 ± 2.6	< 0.001

*PRTEE*, Patient-Related Tennis Elbow Evaluation; *qDASH*, quick Disabilities of the Arm, Shoulder and Hand. *Difference between values for each case at 3-month follow-up and pre-injection was calculated for both scores in both groups. A new variable (paired difference) for which the mean and SD were calculated; **paired difference/score pre-injection. Pairwise comparisons were done by multiple paired *t* tests with a Bonferroni correction to keep the type I error at 5% overall

At final follow-up, the outcomes in the treatment group were excellent in 51 patients (85%) and good in 9 patients (15%), while in the control group, the outcomes were excellent in five patients (8%), good in 19 patients (32%) and poor in 36 patients (60%) (Table 4). Regarding the patients’ satisfaction, 95% were satisfied (either completely or with some reservations) in the treatment group against 25% in the control group (Table 5). Forty patients in the treatment group experienced mild post-injection pain, which resolved completely within three to seven days. Otherwise, there were no other adverse effects related to the procedure.

**Table 4:** Outcome of the patients among both groups at the final follow-up

	Excellent	Good	Poor	Total	*P* value
Treatment group, *n* (%)	51 (85)	9 (15)	0 (00)	60 (100)	< 0.001
Control group, *n* (%)	5 (08)	19 (32)	36 (60)	60 (100)	
Total, *n* (%)	56 (47)	28 (23)	36 (30)	120 (100)	

Fisher’s exact test was used

**Table 5:** Patients’ satisfaction among both groups at the final follow-up

	Completely satisfied	Satisfied with some reservations	Satisfied with important reservations	Dissatisfied	*P* value
Treatment group, *n* (%)	46 (77)	11 (18)	3 (5)	0 (00)	< 0.001
Control group, *n* (%)	5 (8)	10 (17)	8 (13)	37 (62)	

Fisher’s exact test was used

## Discussion

Tennis elbow is a common problem facing orthopaedic surgeons with paucity of scientific rationale to support most of the available treatment modalities [14]. Although resolution of symptoms might occur in 70 to 80% of patients within one year even without treatment [24], chronic elbow tendinopathy might occur with limitation of function and/or activities of daily living [3, 4, 25].

The most commonly injectable materials in treatment of tennis elbow are steroids and PRP [26]. The role of a local steroid is debatable because it acts by suppressing the inflammatory process that is not a consistent part of the pathology [14]. Local steroid injection was reported to give partial and temporary improvement [5]. Moreover, post-injection relapses and recurrences tend to be high due to permanent degenerative changes potentiated by steroids within the tendon substance and due to the associated premature arm overuse secondary to rapid pain relief induced by these injections [5, 27].

There are considerable controversies regarding local injection of PRP in tennis elbow patients. Despite several studies with satisfactory outcomes after using autologous PRP [1, 14, 23], Palacio et al. [28] did not find statistical evidence of better results after autologous PRP compared to corticosteroids or local anaesthetic. Montalvan et al. [29] reported that autologous PRP injection was not more effective than saline injection, after six and 12-month follow-up. In a systematic review, De Vos et al. [30] reported that there was no significant effect of PRP when compared to corticosteroids, saline, autologous whole blood or local anaesthesia. They eventually concluded that there is strong evidence that autologous PRP injection is not even effective in tennis elbow treatment.

Variations in PRP preparation technique can considerably affect the outcomes. The platelet concentration varies by the blood volume taken from the patient [31]. Higher platelets and growth factors concentration need large blood volume. This may not be suitable in comorbid elderly patients or patients using antiplatelet medications [32]. In addition, alterations in centrifuge speed and braking mechanisms may lead to premature platelet activation. Also, presence of concentrated white blood cells may paradoxically induce inflammation with tissue matrix degradation, slow the repair process and induce excessive fibrosis [33, 34]. Moreover, adding platelet-activating agents to PRP preparation may induce coagulopathies or severe pain that may last for few days [33, 34]. Most of the released growth factors have short half-lives (minutes to few hours). If not used within few hours, substantial loss of bioactivity and consequent poor outcome may occur [9].

The L-GF vial is not a true PRP preparation. It contains multiple highly concentrated growth factors that are regulated for the temperature, centrifugation speed, techniques of separation and processing with long-term and sustained release [35]. In vitro platelets stimulation to free growth factors from the alpha granules avoids using platelet-activating agents.

The available clinical trials using allogeneic growth factors in orthopaedic disorders are scarce. Kandil et al. evaluated the efficacy and safety of L-GF in treating 150 plantar fasciitis cases, and reported significant improvement in visual analogue scale (VAS) and Foot Function Index–Revised short form (FFI-Rs) scores and 92% of patients were satisfied [10]. Elgohary et al. [9] reported that L-GF had shown encouraging results and were well tolerated in treatment of symptomatic knee osteoarthritis.

To our knowledge, this is the first study evaluating the efficacy of allogeneic L-GF in tennis elbow treatment. There was significant improvement in PRTEE and qDASH scores and excellent to good outcomes without any significant adverse effects.

However, the control group patients reported pain reduction and improved function over time. This might be due to the natural resolution of the symptoms (being essentially a self-limited disease) [36], placebo effect or the injection procedure which may be beneficial because of the bleeding from forcing fluid at high pressures through tissue planes during injection [14, 37].

The strength points in this study are the prospective randomized controlled design, homogenous population (tennis elbow without previous local injection or surgery), reasonable number of patients and sufficient follow-up period. In addition to the subjective outcomes with patients’ satisfaction assessment, the clinician assessment and changes in PRTEE and qDASH scores were also evaluated.

However, this study has some limitations. For the process of randomization, sealed envelopes with potential bias were used rather than random number generator by computer software. To decrease the potential bias, the envelopes received numbers in advance and were opened sequentially only after the participant’s name was written on the appropriate envelope. The injection site was allocated by digital palpation of the most tender area, not through ultrasonographic guidance. Finally, lack of investigators blinding is limitation with a risk of experimenter bias. Yet, this is not a major limitation because the functional outcomes were essentially evaluated through patients’ determined scores that are not to be influenced by this kind of blinding.

## Conclusions

This prospective controlled study suggests that local injection of allogeneic platelet-derived growth factors is a promising and safe option for treating tennis elbow with significant pain relief, functional improvement and patient satisfaction. Yet, additional studies with larger sample sizes are needed to emphasize these conclusions and assess the durability of these outcomes.

## Data Availability

The datasets generated during and/or analysed during the current study are available from the corresponding author on reasonable request.

## References

[1] Gupta SKV, Bandari D (2016). Autologous platelet-rich plasma injection in tennis elbow and plantar fasciitis. Curr Orthop Pract.

[2] Gautam VK, Verma S, Batra S, Bhatnagar N, Arora S (2015). Platelet-rich plasma versus corticosteroid injection for recalcitrant lateral epicondylitis: clinical and ultrasonographic evaluation. J Orthop Surg.

[3] Walker-Bone K, Palmer KT, Reading IC, Coggon D, Cooper C (2012). Occupation and epicondylitis: a population-based study. Rheumatology (Oxford).

[4] Buchbinder R, Richards BL (2010). Is lateral epicondylitis a new indication for botulinum toxin?. CMAJ.

[5] Smidt N, van der Windt DA, Assendelft WJ, Devillé WL, Korthals-de Bos IB, Bouter LM (2002). Corticosteroid injection, physiotherapy, or wait-and-see policy for lateral epicondylitis: a randomised controlled trial. Lancet.

[6] Olaussen M, Holmedal Ø, Lindbaek M, Brage S (2009). Physiotherapy alone or in combination with corticosteroid injection for acute lateral epicondylitis in general practice: a protocol for a randomised, placebo-controlled-study. BMC Musculoskelet Disord.

[7] Galvin R, Callaghan C, Chan WS, Dimitrov BD, Fahey T (2011). Injection of botulinum toxin for treatment of chronic lateral epicondylitis: systematic review and meta-analysis. Semin Athritis Rheum.

[8] Lindenhovius A, Henket M, Gilligan BP, Lozano-Calderon S, Jupiter JB, Ring D (2008). Injection of dexamethasone versus placebo for lateral elbow pain: a prospective, double-blind, randomized clinical trial. J Hand Surg Am.

[9] Elgohary R, Diab A, Elgndy H, Fahmy H, Gado K (2019). Evaluating the effectiveness of intra-articular knee injection using allogenic platelet derived lyophilized growth factors in Egyptian patients with symptomatic primary knee osteoarthritis. Ann Rheum Dis.

[10] Kandil MI, Tabl EA, Elhammady AS (2020). Prospective randomized evaluation of local injection of allogeneic growth factors in plantar fasciitis. Foot Ankle Int.

[11] Othman AMA (2014). Treatment of chronic lateral epicondylitis: platelet rich plasma versus extra-corporeal shock wave therapy. Open J Orthop.

[12] Alsousou J, Thompson M, Hulley P, Noble A, Willett K (2009). The biology of platelet-rich plasma and its application in trauma and orthopaedic surgery: a review of the literature. J Bone Joint Surg Br.

[13] Yadav R, Kothari SY, Borah D (2015). Comparison of local injection of platelet rich plasma and corticosteroids in the treatment of lateral epicondylitis of humerus. J Clin Diagn Res.

[14] Reddy VV, Chandru V, Patel I, Gopalakrishna SV (2016). Comparison between corticosteroid, platelet rich plasma (PRP) and xylocaine infiltration for lateral epicondylosis (tennis elbow): a prospective randomized study. J Trauma Treat.

[15] Andres BM, Murrell GAC (2008). Treatment of tendinopathy: what works, what does not, and what is on the horizon. Clin Orthop Relat Res.

[16] Cazzell S, Stewart J, Agnew PS (2018). Randomized controlled trial of micronized dehydrated human amnion/ chorion membrane (dHACM) injection compared to placebo for the treatment of plantar fasciitis. Foot Ankle Int.

[17] Akbarzadeh S, McKenzie MB, Rahman MM, Cleland H (2021). Allogeneic platelet-rich plasma: is it safe and effective for wound repair?. Eur Surg Res.

[18] Pan L, Yong Z, Yuk KS, Hoon KY, Yuedong S, Xu J (2016). Growth factor release from lyophilized porcine platelet-rich plasma: quantitative analysis and implications for clinical applications. Aesth Plast Surg.

[19] Schulz KF, Altman DG, Moher D (2010). CONSORT 2010 statement: updated guidelines for reporting parallel group randomised trials. BMJ.

[20] Rompe JD, Overend TJ, MacDermid JC (2007). Validation of the patient-related tennis elbow evaluation questionnaire. J Hand Ther.

[21] Gummesson C, Ward MM, Atroshi I (2006). The shortened disabilities of the arm, shoulder and hand questionnaire (Quick DASH): validity and reliability based on responses within the full-length DASH. BMC Musculoskelet Disord.

[22] Gosens T, Peerbooms JC, van Laar W, den Oudsten BL (2011). Ongoing positive effect of platelet-rich plasma versus corticosteroid injection in lateral epicondylitis: a double-blind randomized controlled trial with 2-year follow-up. Am J Sports Med.

[23] Peerbooms JC, Sluimer J, Bruijn DJ (2010). Positive effect of an autologous platelet concentrate in lateral epicondylitis in a double-blind randomized controlled trial: platelet-rich plasma versus corticosteroid injection with a 1-year follow-up. Am J Sports Med.

[24] Tonks JH, Pai SK, Murali SR (2007). Steroid injection therapy is the best conservative treatment for lateral epicondylosis: a prospective randomized controlled trial. Int J Clin Pract.

[25] Silverstein B, Adams D (2007) Work-related musculoskeletal disorders of the neck, back, and upper extremity in Washington State. Olympia, Washington: SHARP Program, Washington State Department of Labor and Industries 1–98. https://lni.wa.gov/safety-health/safety-research/files/2007/2007WmsdRpt.pdf. Accessed 10 June 2021

[26] Bjordal JM, Couppe C, Ljunggren AE (2001). Low level laser therapy for tendinopathy: evidence of a dose response. Phys Ther Rev.

[27] Edwards SG, Calandruccio JH (2003). Autologous blood injections for refractory lateral epicondylitis. J Hand Surg Am.

[28] Palacio EP, Schiavetti RR, Kanematsu M, Ikeda TM, Mizobuchi RR, Galbiatti JA (2016). Effects of platelet-rich plasma on lateral epicondylitis of the elbow: prospective randomized controlled trial. Rev Bras Ortop.

[29] Montalvan B, Le Goux P, Klouche S, Borgel D, HardyP BM (2016). Inefficacy of ultrasound-guided local injections of autologous conditioned plasma for recent epicondylitis: results of a double-blind placebo-controlled randomized clinical trial with one-year follow-up. Rheumatology (Oxford).

[30] De Vos RJ, Windt J, Weir A (2014). Strong evidence against platelet-rich plasma injections for chronic lateral epicondylar tendinopathy. A systematic review. Br J Sports Med.

[31] Steven AP, Oscar C, Yenern Y (2010). On the horizon from ORS. JAAOS.

[32] Nguyen RT, Borg-Stein J, McInnis K (2011). Applications of platelet-rich plasma in musculoskeletal and sports medicine: an evidence-based approach. PM R.

[33] Zhang ZY, Huang AW, Fan JJ, Wei K, Jin D, Chen B (2013). The potential use of allogeneic platelet-rich plasma for large bone defect treatment: immunogenicity and defect healing efficacy. Cell Transplant.

[34] Pretorius E, Briedenhann S, MarxJ SE, Merwe CVD, Pieters M, Franz C (2007). Ultrastructural comparison of the morphology of three different platelet and fibrin fiber preparations. Anat Rec.

[35] Sonker A, Dubey A, Bhatnagar A, Chaudhary R (2015). Platelet growth factors from allogeneic platelet-rich plasma for clinical improvement in split-thickness skin graft. Asian J Transfus Sci.

[36] Jindal N, Gaury Y, Banshiwal RC, Lamoria R, Bachhal V (2013). Comparison of short term results of single injection of autologous blood and steroid injection in tennis elbow: a prospective study. J Orthop Surg Res.

[37] Ansari MAQ, Shah SA, Jidgekar SR (2016). Tennis elbow—efficacy of local corticosteroid injection in its management. IOSR J Sports Phys Educ (IOSR-JSPE).

